# Pulmonary Pleomorphic Carcinoma Detected as a Result of Pneumothorax and the Subsequent Occurrence of Multiple Cystic Metastases

**DOI:** 10.1155/2014/219273

**Published:** 2014-08-20

**Authors:** Hideaki Yamakawa, Masahiro Yoshida, Masami Yabe, Yuri Baba, Emiri Baba, Hiroaki Katagi, Takeo Ishikawa, Masamichi Takagi, Takeo Nakada, Tadashi Akiba, Kazuyoshi Kuwano

**Affiliations:** ^1^Department of Internal Medicine, Division of Respiratory Medicine, Jikei University School of Medicine, Kashiwa Hospital, 163-1 Kashiwashita, Kashiwa, Chiba, Japan; ^2^Department of Internal Medicine, Division of Diagnostic Pathology, Jikei University School of Medicine, Kashiwa Hospital, Chiba, Japan; ^3^Department of General Thoracic Surgery, Division of Surgery, Jikei University School of Medicine, Kashiwa Hospital, Chiba, Japan; ^4^Department of Internal Medicine, Division of Respiratory Medicine, Jikei University School of Medicine, Tokyo, Japan

## Abstract

A 39-year-old man was admitted for spontaneous pneumothorax. He underwent pulmonary resection to correct the lesion causing the air leakage, and a pathological diagnosis of pulmonary pleomorphic carcinoma was made because we thought that the pneumothorax developed due to the direct rupture of necrotic neoplastic tissue into the pleural cavity. After the operation, the patient received chemotherapy, during which multiple cystic metastases gradually developed in the lung that caused repeated occurrences of pneumothorax. Clinicians must be careful to recognize that pneumothorax can also be a complication of primary and various metastatic pulmonary malignancies.

## 1. Introduction

Primary spontaneous pneumothorax occurs because of the rupture of subpleural blebs, whereas secondary spontaneous pneumothorax refers to the development of pneumothorax due to the evolution of pulmonary pathology. Although secondary pneumothorax is most often associated with bulbous emphysema, parenchymal lung diseases also often lead to this complication. Malignancy involving the lung is one such disease process leading to secondary spontaneous pneumothorax [1, 2]. However, multiple thin-walled cystic metastases in the lung are extremely rare [3]. We report our experience of a rare case of a patient diagnosed as having pulmonary pleomorphic carcinoma that caused pneumothorax. During chemotherapy, pneumothorax occurred repeatedly due to cystic metastases, which is an uncommon manifestation.

## 2. Case Report

**Figure 1:**
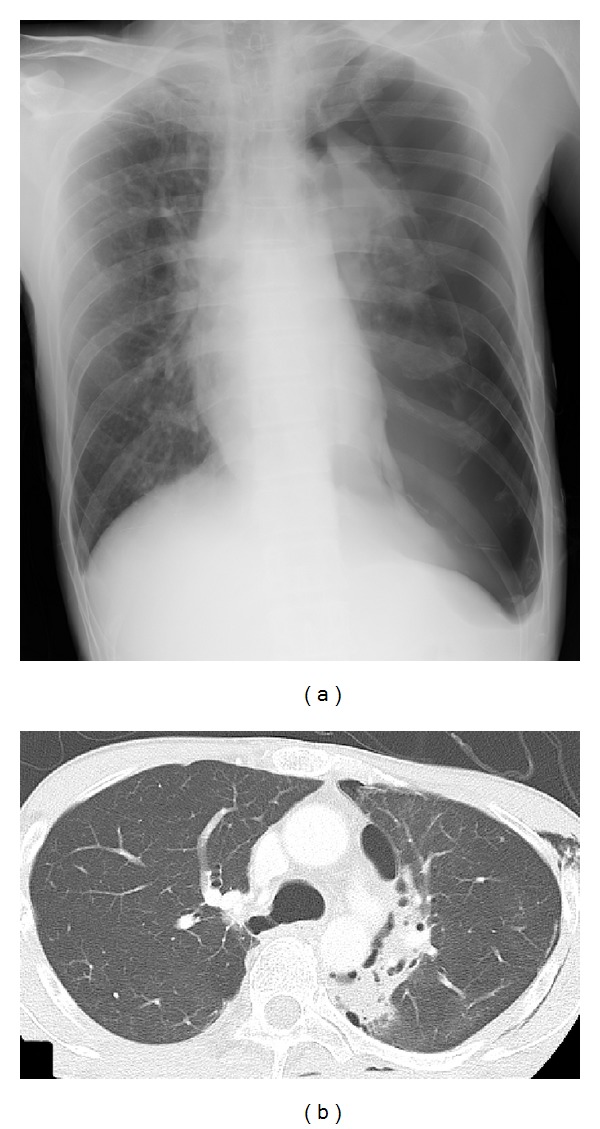
Chest X-ray performed at initial presentation and chest computed tomography on the 11th hospital day. (a) On admission, chest X-ray showed a left-sided pneumothorax. (b) On the 11th hospital day, chest computed tomography showed intrapulmonary infiltration on the mediastinal side of the left upper lobe, and no emphysematous changes were seen.

**Figure 2:**
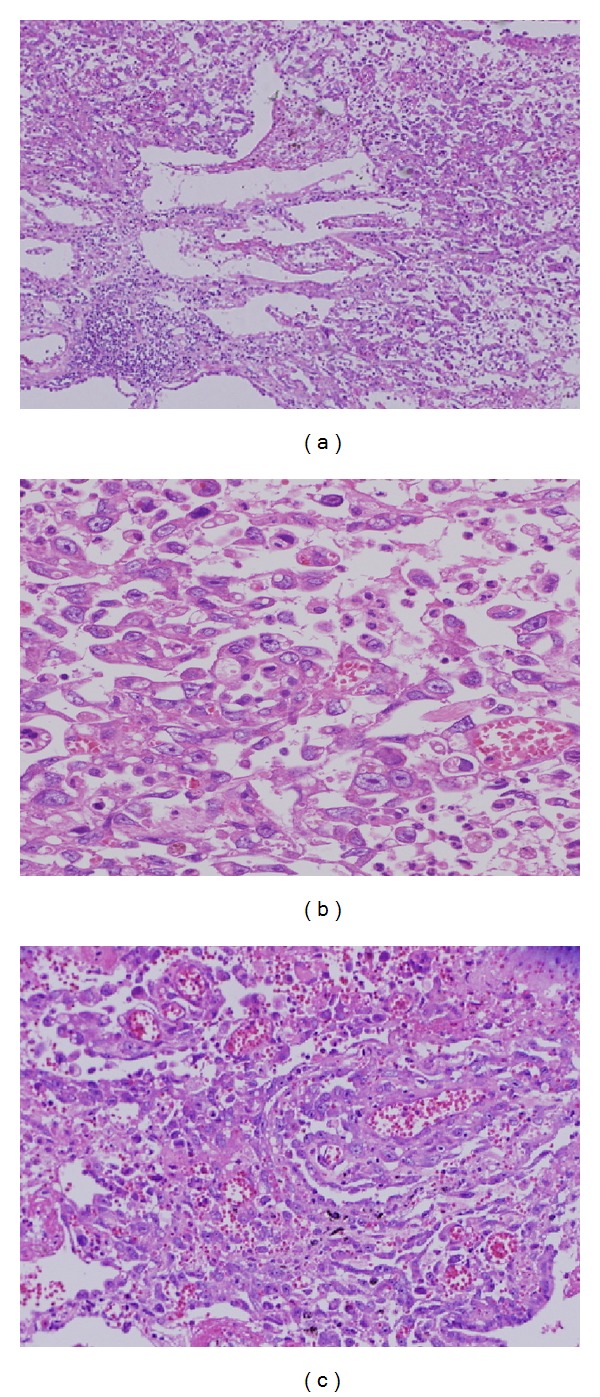
Microscopic findings of the tumor show mainly packed atypical pleomorphic spindle cells with giant cells ((a) hematoxylin and eosin stain, ×40; (b) ×400) and partial papillary adenocarcinoma ((c) ×100).

**Figure 3:**
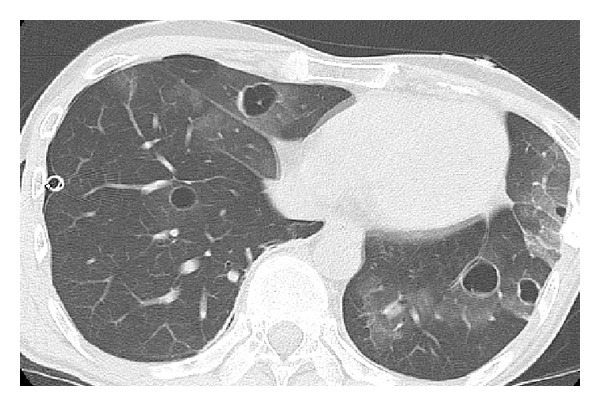
Chest computed tomography taken 9 months after the operation for pulmonary complications shows bilateral thin-walled cystic lesions that are surrounded by ground glass opacities, for which the patient underwent placement of a drainage tube in the pleural space for right pneumothorax.

The patient was a 39-year-old Japanese man with a history of treatment at the age of 20 years for Hodgkin's lymphoma that underwent complete remission with no recurrence. From February 2013, he developed a dry cough and sputum production. Afterwards, he occasionally had bloody sputum and dyspnea and was referred to our hospital in June 2013. A chest X-ray on admission showed left pneumothorax (Figure 1(a)). He underwent drainage of the pleural space, but lung expansion was poor, and the air leakage was not improved. On the 11th hospital day, a contrast-enhanced computed tomography (CT) scan revealed intrapulmonary infiltration in the mediastinal side of the left upper lobe (Figure 1(b)). There was no swelling of the hilar or mediastinal lymph nodes, and no emphysematous changes were seen. Therefore, he underwent surgery on the 13th hospital day. Although we were not able to confirm the site of the air leakage clearly, we recognized a high amount of sphacelus tissue adhering to neighboring tissue in the left upper lobe (S1+2) and lower lobe (S6). We therefore considered these lesions to be the cause of the air leakage, and we resected the left upper lobe and S6. Histological findings of these lesions showed prominent proliferation of elements of malignant giant cells and spindle cells with small foci of adenocarcinoma, and a pathological diagnosis of primary pulmonary pleomorphic carcinoma was made, with the margin described as positive at the edge of the bronchus (Figure 2). After the operation, the patient received chemotherapy (1st line: carboplatin and paclitaxel, 2nd line: docetaxel, and 3rd line: pemetrexed). However, he repeatedly complained of bloody sputum, and chest CT revealed multiple cystic metastases in the lung. Moreover, during chemotherapy, pneumothorax occurred three times after the direct rupture into the pleural cavity of developing cystic lesions in the pulmonary metastatic tissue (Figure 3). His condition deteriorated and he died 10 months after surgery.

## 3. Discussion

We describe a case of pulmonary pleomorphic carcinoma complicated by pneumothorax that occurred repeatedly due to cystic metastases in the lung. Pneumothorax appears to be quite rare in lung cancer patients. Steinhäuslin and Cuttat reported that among patients presenting with spontaneous pneumothorax, only 1.8% of patients had lung cancer [4]. Pneumothorax has been reported as the first sign of lung involvement for certain tumors as a complication arising from radiotherapy or cytotoxic chemotherapy in patients suffering from a variety of malignancies [5, 6]. In another report, pneumothorax was found in only 0.32% of primary lung cancer patients. Moreover, of these patients, pneumothorax was the initial manifestation of primary lung cancer in 16.7% and occurred as a complication in another 83.3% of patients [7].

Pneumothorax occurring in lung cancer patients may be caused by direct rupture of necrotic tissue into the pleural cavity or rupture of a subpleural bleb, by the formation of interstitial air due to partial bronchial obstruction by the tumor, by complications arising from radiation therapy and cytotoxic chemotherapy, or by any combination of these factors [7]. In our patient, a tumor with necrotic tissue and hematoma was exposed on the pleural surface, and therefore, we thought that the pneumothorax developed following the direct rupture of this necrotic neoplastic tissue into the pleural cavity. Moreover, our patient suffered recurrent pneumothorax that was thought to be related to the rupture of cystic metastases into the pleural cavity. Metastatic soft tissue sarcomas or primary bronchioloalveolar carcinoma may show cystic metastasis, but they are extremely rare [8, 9]. To our knowledge, this finding has not previously been reported in association with pulmonary pleomorphic carcinoma. Imai et al. reported an autopsy case of bronchioloalveolar carcinoma accompanied by multiple pulmonary cysts and suggested that extension of tumor cells along alveolar walls destroyed the septa to form centrilobular emphysematous cysts [9]. Hasegawa et al. reported a case of pulmonary cysts due to metastatic soft tissue sarcoma and speculated that microscopic cavitary metastases within normal lung parenchyma represented an early stage of macroscopic cystic lesions and that a check-valve mechanism was responsible for their formation [8]. We did not investigate the cystic lesions in our patient pathologically. Therefore, further investigation into cystic formation will be necessary.

Pulmonary pleomorphic carcinoma is rare, comprising only 0.1–0.4% of all pulmonary malignancies according to previous reports [10]. According to the 2004 World Health Organization (WHO) histologic classification, pleomorphic carcinoma is the most common subtype of pulmonary sarcomatoid carcinoma. In pleomorphic carcinoma, the sarcomatoid component includes spindle or giant cells, alone or variably admixed, whereas the epithelial component is composed of conventional nonsmall cell lung carcinomas (NSCLCs) featuring adenocarcinoma, squamous cell carcinoma, or undifferentiated large cell carcinoma [11]. The prognosis for pulmonary pleomorphic carcinoma is generally thought to be worse than that for conventional NSCLC due to its resistance to chemotherapy and radiotherapy and early metastases that form within a few months in organs such as the brain, bone, and adrenal gland and in unusual sites, including the esophagus, jejunum, rectum, and kidney [12].

The prognosis for primary lung cancer complicated by pneumothorax is poor. Steinhäuslin and Cuttat reported that the occurrence of a pneumothorax neither alters the treatment of the underlying disease nor modifies the 1-year prognosis. Five-year survival is nil, suggesting that lung cancers accompanied by pneumothorax are at an advanced stage of the disease [4]. Another report indicated that pneumothorax is an ominous sign: most lung cancer patients with pneumothorax died within 3 months [7]. The poor outcome of such patients may be attributed to the extensive lung damage caused by the tumor itself and/or the antitumor therapy and the deleterious effect of the pneumothorax on pulmonary function reserve. The clinical course of the bilateral pulmonary pleomorphic cancer complicated by pneumothorax in our patient was very poor.

Several previous reports showed that the histologic types of primary lung malignant disease complicated by pneumothorax include adenocarcinoma, squamous cell carcinoma, large call carcinoma, and sarcoma [4, 7, 13–15]. However, the number of case reports of patients with pulmonary pleomorphic carcinoma increased after the disease was formally placed in the WHO histologic classification, and reports of the tendency of this disease toward necrosis and chest wall invasion are known to be more frequent [16]. Therefore, further study is necessary to determine whether pulmonary pleomorphic carcinoma tends to cause rupture into the thoracic cavity and subsequent pneumothorax as in our patient.

We described a case of pulmonary pleomorphic carcinoma detected as a result of pneumothorax, which occasionally may be the initial manifestation of lung cancer. Moreover, our patient suffered recurrent pneumothorax due to the rupture of cystic metastases. Therefore, clinicians must be careful to recognize that pneumothorax can also be a complication of primary and various metastatic pulmonary malignancies.
